# The Additional Role of F18-FDG PET/CT in Characterizing MRI-Diagnosed Tumor Deposits in Locally Advanced Rectal Cancer

**DOI:** 10.3390/tomography10040048

**Published:** 2024-04-22

**Authors:** Mark J. Roef, Kim van den Berg, Harm J. T. Rutten, Jacobus Burger, Joost Nederend

**Affiliations:** 1 Department of Radiology and Nuclear Medicine, Catharina Hospital Eindhoven, 5623 EJ Eindhoven, The Netherlands; joost.nederend@catharinaziekenhuis.nl; 2 Catharina Cancer Institute, Catharina Hospital Eindhoven, 5623 EJ Eindhoven, The Netherlands; kim.vd.berg@catharinaziekenhuis.nl; 3 Department of Surgery, Catharina Hospital Eindhoven, 5623 EJ Eindhoven, The Netherlands; harm.rutten@catharinaziekenhuis.nl (H.J.T.R.); pim.burger@catharinaziekenhuis.nl (J.B.)

**Keywords:** locally advanced rectal cancer, F18-FDG PET/CT, SUV measurements, partial volume, spill-in

## Abstract

**Rationale:** F18-FDG PET/CT may be helpful in baseline staging of patients with high-risk LARC presenting with vascular tumor deposits (TDs), in addition to standard pelvic MRI and CT staging. **Methods:** All patients with locally advanced rectal cancer that had TDs on their baseline MRI of the pelvis and had a baseline F18-FDG PET/CT between May 2016 and December 2020 were included in this retrospective study. TDs as well as lymph nodes identified on pelvic MRI were correlated to the corresponding nodular structures on a standard F18-FDG PET/CT, including measurements of nodular SUVmax and SUVmean. In addition, the effects of partial volume and spill-in on SUV measurements were studied. **Results:** A total number of 62 patients were included, in which 198 TDs were identified as well as 106 lymph nodes (both normal and metastatic). After ruling out partial volume effects and spill-in, 23 nodular structures remained that allowed for reliable measurement of SUVmax: 19 TDs and 4 LNs. The median SUVmax between TDs and LNs was not significantly different (*p* = 0.096): 4.6 (range 0.8 to 11.3) versus 2.8 (range 1.9 to 3.9). For the median SUVmean, there was a trend towards a significant difference (*p* = 0.08): 3.9 (range 0.7 to 7.8) versus 2.3 (range 1.5 to 3.4). Most nodular structures showing either an SUVmax or SUVmean ≥ 4 were characterized as TDs on MRI, while only two were characterized as LNs. **Conclusions:** SUV measurements may help in separating TDs from lymph node metastases or normal lymph nodes in patients with high-risk LARC.

## 1. Introduction

Several MRI-based tumor characteristics of patients diagnosed with locally advanced rectal cancer (LARC) are associated with a relatively poor prognosis: mesorectal fascia involvement (MRF+), grade 4 extramural venous invasion (mrEMVI), tumor deposits (TD) and/or enlarged lateral (extramesorectal) lymph nodes with a short axis of ≥7 mm (LLN) [1]. The existing literature reports rates of local recurrence (LR) and distant metastases (DM) to be approximately 20% and 50%, respectively, for this specific subset of patients, termed high-risk (hr-) LARC [2,3]. In comparison, LR and DM rates for patients with locally advanced rectal cancer who do not possess these unfavorable MRI features fluctuate between 5–10% and 25–40%, respectively [4,5,6,7]. Despite the grim outlook for patients diagnosed with hr-LARC, the current standard treatment approach remains consistent with that for LARC patients who do not exhibit these poor prognostic features. Both groups typically consist of some form of neoadjuvant treatment followed by surgical intervention.

The presence of mrEMVI as an important risk factor in patients with LARC has gained special interest [8]. A meta-analysis performed in 2017 demonstrated an mrEMVI prevalence ranging from 19.8% to 57.4% in patients diagnosed with LARC [9]. A comparison of patients with mrEMVI-positive and -negative LARC at baseline showed an association with synchronous metastases (odds ratio (OR) 5.68, 95% Confidence Interval (CI) 3.75–8.61). Moreover, metachronous metastases were found 3.91 times more frequently in mrEMVI-positive patients (95% CI 2.61–5.86). A retrospective analysis performed by our own study group found a prevalence of mrEMVI of 58.8% in patients with cT3-4 rectal cancer (*n* = 277). TDs were present in 56.4% of the patients with mrEMVI compared to 9.6% of the patients without mrEMVI (*p* < 0.001) [10].

Patients with mrEMVI at baseline are more likely to also have TDs (OR 2.51, 95% CI 2.27–2.77) according to a meta-analysis performed in 2017 [11]. TDs have been recognised as accumulations of malignant cells without the presence of residual lymph node tissue [1]. Both overall survival (OS) and DFS are favorable in patients without TDs compared to patients with TDs at baseline MRI (OS: HR 2.54, 95% CI 1.95–3.30; DFS: HR 2.30, 95% CI 1.82–2.92) [8].

The identification of tumor deposits (TDs) on MRI carries significant prognostic implications and necessitates differentiation from normal lymph nodes and lymph node metastases. In the MRI study conducted by Lord et al., the inter-observer agreement for mesorectal TDs yielded kappa values of 0.77 and 0.83 for the first and second observers, respectively [8]. Another MRI study reported a kappa value of 0.734 [11]. While these kappa values suggest an acceptable degree of inter-observer agreement, they fall short of being perfect, thus highlighting the need for supplementary imaging techniques. We hypothesized that F18-FDG PET/CT imaging could serve as a valuable adjunct in distinguishing TDs from normal lymph nodes and lymph node metastases by measuring their standardized uptake values (SUVs).

F18-FDG PET/CT plays only a limited role in the staging of LARC however. It has been proven valuable in patients with resectable liver metastases in search of extrahepatic metastatic spread that would preclude surgery. F18-FDG PET/CT has been used for monitoring (neoadjuvant) therapies such as chemoradiotherapy, both in LARC patients eligible for surgery as well as wait-and-see strategies [12]. In addition to this, our study group has implemented F18-FDG PET/CT in monitoring neoadjuvant induction chemotherapy in high-risk LARC as well, giving the opportunity to compare baseline F18-FDG PET/CT to pelvic MRI. In F18-FDG PET/CT, more aggressive tumor behaviour has been shown to be associated with more intense FDG uptake, which can be quantified by measuring SUVs [13]. Although this has been proven for several primary tumors including rectal carcinoma, it is currently unknown whether this also holds for TDs versus lymph nodes. Therefore, it was hypothesized that TDs of a particular size would exhibit higher SUV values compared to normal lymph nodes or lymph node metastases of equivalent size.

However, SUV measurements of these often relatively small mesorectal nodular structures are not always straightforward because of confounding effects of partial volume (spill-out) and spill-in. Partial volume effects are thought to play an increasingly important role when the nodular structures become smaller than around twice the spatial resolution of the imaging system (FWHM), causing incorrect low SUV values [14]. Spill-in effects (shine through effects) work the other way by incorrectly increasing the SUV values of nodular structures close to tumors or organs with high SUV values, e.g., bladder contents. In theory, partial volume effects and spill-in effects can cancel each other out, causing apparently correct SUV values, but this is highly unreliable [15]. Therefore, taking into account these possibly confounding effects of partial volume and spill-in, it was anticipated that no differences in SUV values between TDs and lymph nodes (both normal and metastatic) would be found when small and/or close to the primary tumor or bladder contents with high SUV values. However, for larger TDs and lymph nodes at a greater distance from the primary tumor or bladder contents, the hypothesis may hold that TDs of a certain size have higher SUV values than normal lymph nodes or lymph node metastases of the same size. In order to test our hypothesis, it was therefore decided not only to measure the SUV of mesorectal nodular structures that were identified as TDs or lymph nodes (both normal and metastatic) on MRI, but also the size and distance to the nearest organ with high SUV values, possibly causing spill-in.

## 2. Materials and Methods

### 2.1. Study Setting

This study was conducted in a tertiary care teaching hospital. Informed consent was waived because of the retrospective nature of the study (Medical research Ethics Committees United, Nieuwegein, The Netherlands, registration nr. W23.008). Rectal cancer patients that had their scans between May 2016 and December 2020 were included. Patients were selected from an existing surgical and non-surgical database.

### 2.2. Patients

All patients with locally advanced rectal cancer that had TDs on their baseline MRI of the pelvis and had a baseline F18-FDG PET/CT were included in this retrospective study. The time interval between MRI and F18-FDG PET/CT was on average 17 days (range 3 to 47 days).

### 2.3. Radiologic Assessment

#### 2.3.1. MRI

The MRI scans of all included patients were re-evaluated by an experienced radiologist with specific expertise in rectal cancer and trained in the assessment of mrEMVI and TD. The radiologist was blinded to patient characteristics and follow-up data. At least T2-weighted images in the sagittal and transversal planes of sufficient quality and with a slice thickness between 3 and 5 mm had to be available. In some cases, the MRI images were imported from the referring hospital.

Special attention was given to the occurrence of mrEMVI and TD. mrEMVI was evaluated for the vascular invasion grade: grade 2 to 4. TDs were defined as irregular, nodule-like structures in line with a vessel without the typical characteristics of a lymph node, as described by Lord et al. [8]. The sizes of all nodular structures were measured on MRI.

#### 2.3.2. F18-FDG PET/CT

Whole-body images from skull base to midthigh were obtained by PET/CT (Discovery 710, GE Healthcare, Milwaukee, WI, USA) in accordance with accepted institutional procedures. Patients were asked to void before image acquisition was started approximately 60 min after tracer injection.

#### 2.3.3. Correlating MRI and F18-FDG PET/CT Images

In a consensus reading, the radiologist and an experienced nuclear physician correlated the thus identified TDs and lymph nodes on pelvic MRI to the corresponding nodular structures on a standard F18-FDG PET/CT. In addition to size measurements, the nuclear physician measured the SUVmax and SUVmean of all nodular structures as well as the closest distance to organs with high activity (usually the primary tumor but sometimes the bladder) in any of the three imaging planes. When the radiologist and the nuclear physician disagreed, the radiologist was decisive with respect to the MRI determination of the nature of the nodule. The nuclear physician was decisive in correlating the nodular structures on the F18-FDG PET/CT images.

**Figure 1:**
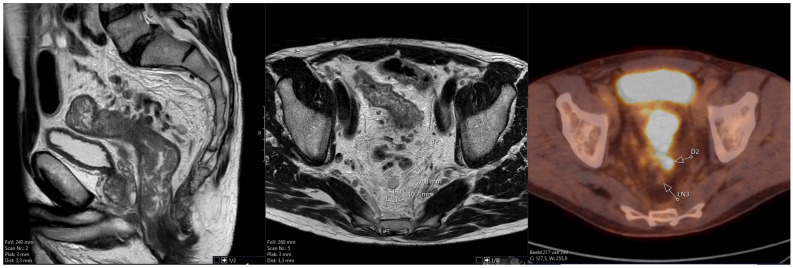
Example of the anonymized combined MRI and F18-FDG PET/CT layout used in this study. The example shows a tumor deposit (designated D2) and a lymph node (designated LN3). Only the primary tumor is depicted on the sagittal image.

Figure 1 shows an example of the anonymized combined MRI and F18-FDG PET/CT layout that was used in this study.

In order to avoid partial volume effects, the minimum size was set at 12 mm taking into account the spatial resolution of about 6 mm FWHM of our system. For SUVmax measurements, the minimum distance was set at 19 mm, thus avoiding spill-in. For SUVmean measurements, no distance was set because SUVmean tends to be less susceptible to spill-in effects [15].

### 2.4. Statistical Analysis

Statistical analyses were performed using the IBM SPSS statistical package, version 24 (IBM, Armonk, NY, USA). A *p*-value < 0.050 was considered significant. Individual variables were compared with Mann–Whitney-U testing for TDs versus LNs: nodal size, distance to the nearest high-activity organ, SUVmax and SUVmean.

## 3. Results

A total number of 320 nodular structures were identified on MRI in 62 patients, 213 TDs and 107 lymph nodes (both normal and metastatic). In 304 of 320 nodular structures on MRI, corresponding nodular structures were identified on F18-FDG PET/CT. Sixteen nodular structures on MRI had no obvious correlation on F18-FDG PET/CT, mostly because of the different angles between both scans. Thus, a total number of 198 TDs was identified as well as 106 lymph nodes (both normal and metastatic). See Table 1 for pelvic MRI and F18-FDG PET/CT imaging details.

**Figure 2:**
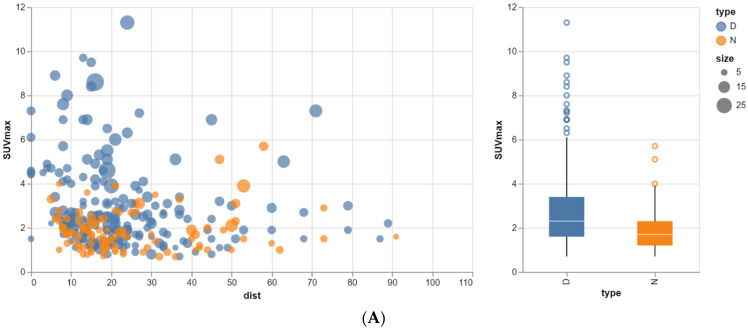

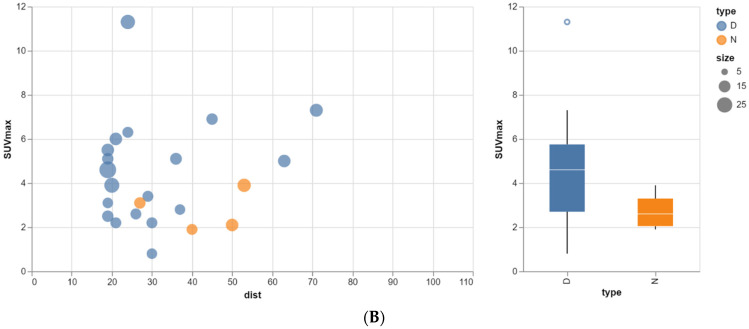
(**A**) Relationship between SUVmax, size and closest distance to high-activity organs for both TDs and LNs. Dist: distance to high-activity organs, D: tumor deposit, N: lymph node. Size and distance both in mm. For all nodular lesions. (**B**) Relationship between SUVmax, size and closest distance to high-activity organs for both TDs and LNs. Dist: distance to high-activity organs, D: tumor deposit, N: lymph node. Size and distance both in mm. For remaining nodular lesions taking into account effects of partial volume and spill-in.

F18-FDG PET/CT changed the N-stage in only three patients: in two patients, it was upstaged from N1a/c to N1c/2a, and in one patient, it was upstaged from N1b/c to N1c/2a. In addition, three patients were upstaged to M1 due to distant lymph node metastases on F18-FDG PET/CT. More lymph node metastases were detected on F18-FDG PET/CT due to the larger field of view. Two patients had mrEMVI grade 2 and 47 patients had mrEMVI grade 3, while 13 patients had mrEMVI grade 4. Six patients had only one TD, 9 patients had 2 TDs, 18 patients had 3 TDs, 10 patients had 4 TDs and 5 patients had 5 TDs, while 14 patients had more than 5 TDs. Of the 198 TDs in total, 65 had a short axis size of 10 mm or more. With respect to lymph nodes (both normal and metastatic LNs), 22 patients had only one LN, 11 had 2 LNs, 9 had 3 LNs, 4 had 4 LNs and 2 had 5 LNs, while another 2 had more than 5 LNs. Twelve patients had TDs only. Only 10 lymph nodes out of 106 in total had a short axis size of 10 mm or more. Figure 2 shows the relationship between SUVmax and the closest distance to high-activity organs for both TDs and LNs. Moreover, the size of the nodular structures is depicted. Figure 2A shows the results for all nodular lesions. Figure 2B shows the results for the remaining nodular lesions taking into account the effects of partial volume and spill-in.

**Figure 3:**
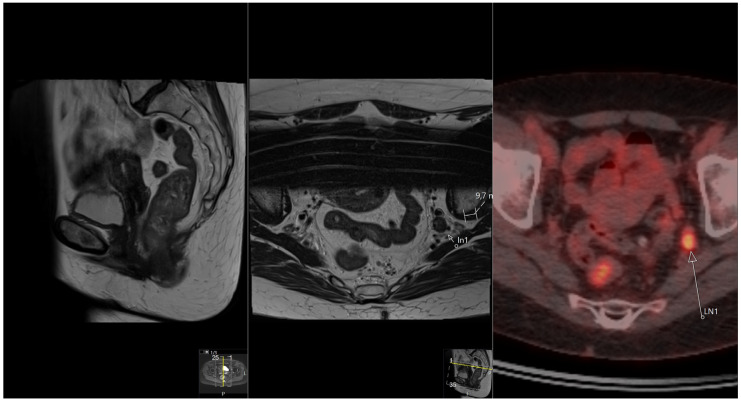
Nodular structure characterized on MRI as lymph node metastasis, located in the left lateral compartment. SUVmax = 5.7. Only the primary tumor is depicted on the sagittal image.

When all nodular structures are taken into account, no differences between TD and LN SUVmax can be found, probably because of interfering partial volume and spill-over effects (Figure 2A). However, most nodular structures showing an SUVmax > 4 were characterized as TDs on MRI, while only two were characterized as LNs. These two lymph nodes could be easily characterized as such because they were located in the lateral compartments. See Figure 3.

Partial volume effects were relevant for the majority of the nodular structures, making the significant difference in size of the TDs versus the LNs less relevant. With respect to the effects of spill-over, there were no significant differences in distance to the nearest high-activity organs between TDs and LNs. See Table 2.

**Figure 4:**
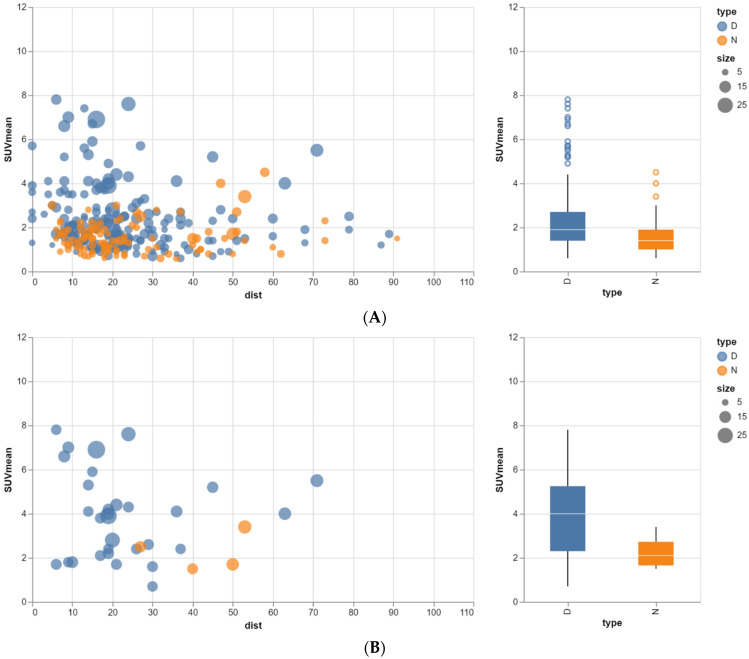
(**A**) Relationship between SUVmean, size and closest distance to high-activity organs for both TDs and LNs. Dist: distance to high-activity organs, D: tumor deposit, N: lymph node. Size and distance both in mm. For all nodular lesions. (**B**) Relationship between SUVmean, size and closest distance to high-activity organs for both TDs and LNs. Dist: distance to high-activity organs, D: tumor deposit, N: lymph node. Size and distance both in mm. For remaining nodular lesions taking into account effects of partial volume and spill-in.

When the minimum size was set at 12 mm in order to avoid partial volume effects and the minimum distance was set at 19 mm, thus avoiding spill-in, only 23 nodular structures remained: 19 TDs and 4 LNs (Figure 2B). The median SUVmax between the designated TDs and LNs is still not significantly different, but there is a trend (*p* = 0.096): 4.6 (range 0.8 to 11.3) versus 2.8 (range 1.9 to 3.9). For the median SUVmean, there is also a trend towards a significant difference (*p* = 0.08): 3.9 (range 0.7 to 7.8) versus 2.3 (range 1.5 to 3.4). See Figure 4. Figure 4A shows the results for all nodular lesions. Figure 4B shows the results for the remaining nodular lesions taking into account the effects of partial volume and spill-in.

## 4. Discussion

This study suggests that SUV measurements may help in separating TDs from lymph node metastases or normal lymph nodes. This may have implications with respect to patient prognosis and possibly also patient management. This study also provides some relevant insights into the confounding effects of partial volume and spill-in, with respect to the SUV measurements of often small mesorectal nodular structures as found in high-risk LARC.

### 4.1. SUV Measurements for Separating TDs from Lymph Node Metastases or Normal Lymph Nodes

On pelvic MRI, the mesorectal nodular structures of high-risk LARC are characterized using predefined size, appearance and location criteria for TDs and lymph node metastases, respectively [8,9]. The mesorectal TDs thus characterized had acceptable but not perfect kappa values of 0.77 and 0.83 for inter-observer agreement between observer 1 and 2, respectively, in the MRI study of Lord et al. [8]. In a second MRI study, a kappa value of 0.734 was observed [11]. To the best of our knowledge, F18-FDG PET/CT has not been used in order to separate TDs from lymph node metastases or normal lymph nodes. Because most of the nodular structures were relatively small and located close to the primary tumor, our hypothesis was only valid in a small number of patients, thus hampering its practical use. Therefore F18-FDG PET/CT cannot be advocated in the standard work-up of LARC for this purpose, even in the subset of high-risk patients. It may be of help in some high-risk LARC patients, however, but nodal structures should be of sufficient size and distance from the primary tumor. Although characterization of the nodular structures by F18-FDG PET/CT had no significant impact on N staging itself, it did make the reader more confident in the characterization of some of the larger nodal lesions. The importance of lymph node staging in LARC has been recognized and has been the subject of several studies [16,17,18,19,20]. SUV measurements have been used to separate lymph node metastases from normal lymph nodes, using different cut-off values for SUVmax in nodes of ≤7 mm and >7 mm, respectively [16]. When using these cut-off values, sensitivity and specificity for lymph node staging (76% and 74%, respectively) are comparable to CT (71% and 67%) and MRI (77% and 71%) [18,19]. In the study of Sung Uk Bae et al., no EMVI or TDs were mentioned, so probably all nodular structures were considered to be lymph nodes [16]. In the study of Alcin et al., venous invasion was mentioned, but TDs were not. SUVmax measurements in lymph nodes were shown to have predictive value for patient survival [19]. With respect to implications for patient management, Ishihara et al. proposed cut-off values for both lymph node size and SUVmax in order to predict lymph node metastases after preoperative chemoradiotherapy and thus selecting the best candidates for pelvic lateral lymph node dissection. In their study, neither EMVI nor TDs were mentioned, but this is perhaps less relevant because EMVI and/or TDs are usually not located in the lateral compartments [20]. Moreover, the two lymph node metastases with SUVmax > 4 in our study could be easily recognized as such because they were located in the lateral compartments.

### 4.2. SUV Measurements and Confounding Effects of Partial Volume and Spill-In

The relatively small size and location close to the primary tumor confound SUV measurements because of the dominant effects of partial volume and spill-in. Two factors that contribute to the partial volume effect (PVE) are the finite spatial resolution of the imaging system (involving scanner hardware, acquisition parameters and reconstruction method) and image sampling on a discrete voxel grid imperfectly matching the actual contours of tracer distribution [21]. The impact of partial volume effects is strongly dependent on lesion size and comes into play when the lesion size falls below two times the resolution of the system, i.e., below 10 to 15 mm for an average PET/CT scanner system [14]. With respect to tracer SUVmax measurements, the partial volume effect will generally result in lower SUVmax values. Several methods to correct for PVE have been developed, from relatively simple recovery coefficients to more complex methods using imaging reconstruction techniques like point spread function (PSF). With respect to colorectal disease, the impact of partial volume correction (PVC) has been studied for both staging and for evaluating response assessment to neoadjuvant therapy. Kawashima et al. describe a PVC method for nodal staging of CRC patients using PSF. The accuracy of nodal staging marginally improved from 89 to 92% [22]. In their study on response assessment, Hatt et al. could not demonstrate any impact of PVC [23]. Because of these somewhat discouraging results, we decided not to apply any PVC method to our uncorrected data but to study contributors to PVE in greater detail as well as its effects on nodal lesion determination.

Both spill-out and spill-in contribute to PVE. Spill-out is the three-dimensional image blurring introduced to the inherent finite spatial resolution of the imaging system. SUV measurements of the radioactive signal in these blurred images usually yield lower values for small nodules. In contrast, spill-in tends to increase nodal SUV measurements because of the signal coming in from adjacent highly radioactive structures like the primary tumor, physiological bowel activity or urinary activity in the bladder. With respect to the pelvis, Akerele et al. studied spill-in effects on F18-FDG SUV measurements in nodal structures located at different distances from the highly radioactive region of the urinary bladder. In this phantom study, spill-in effects were found to be important for SUVmax measurements in nodal structures at a distance not greater than 19 mm from the urinary bladder wall [15]. With respect to SUVmean measurements, spill-in effects were less pronounced, which is the reason why we decided to measure both. Silva-Rodriguez et al. came to the same conclusions in their phantom study using a different radioactive compound F18- FCH, in that SUVmax measurements are more susceptible to spill-in effects than measurements of SUVmean [24]. In an additional phantom study, Akerele et al. showed that in SUVmean measurements of small lesions (6–20 mm), spill-out is always dominant over spill-in. Regarding SUVmax measurements, spill-in becomes dominant over spill-out the closer the nodal structure is located to the highly radioactive region [25]. Based on these results of phantom studies, we supposed spill-in effects to be relevant for nodal SUV measurements at distances up to 19 mm from the primary tumor. In addition to small size, this reduced the number of nodal structures that could be characterized by SUV measurements even more.

### 4.3. Limitations

This single site study has several limitations. The nodular structures in the mesorectum were identified as TDs or lymph nodes using imaging criteria but were not proven as such by histopathological examination, thus lacking the gold standard that MRI by itself cannot replace. This might lead to limitations in the conclusion of the study. The lack of histopathological proof has multiple causes. The main reason is that current guidelines advocate neoadjuvant treatment in patients with locally advanced rectal carcinoma and TDs. Additionally, the patients that do not qualify for surgery because of metastatic spread usually receive chemotherapy and/or radiotherapy for distant and/or local control, respectively. In most of these cases, the nodular structures, being TDs or lymph nodes, respond well to established therapies, leaving no histopathological substrate that can be examined.

With respect to the number of nodular structures in the mesorectum that can be characterized, ruling out confounding effects of partial volume and spill-in on SUV measurements had a great impact. This is even more relevant for measurements of SUVmax than for SUVmean because SUVmean tends to be less susceptible to spill-in effects than SUVmax. This resulted in only 23 remaining nodular structures where SUVmax and 35 where SUVmean could be reliably measured, respectively. Thus, the remaining number of nodular structures after taking into account the effects of partial volume and spill-in is very low, hampering proper statistics. This indicates the need for more patients in a future prospective study.

## 5. Conclusions

The main finding of this study is that F18-FDG PET/CT may be helpful in baseline staging of some patients with high-risk LARC presenting with mesorectal TDs, in addition to standard pelvic MRI and CT staging. SUV measurements may help in separating TDs from lymph node metastases or normal lymph nodes. This study also provides some relevant insights into the confounding effects of partial volume and spill-in, with respect to SUV measurements of often small mesorectal nodular structures as found in high-risk LARC.

## Figures and Tables

**Table 1:** Pelvic MRI and F18-FDG PET/CT imaging details.

Number of patients	62	
Location of the primary tumor in the rectum		
Proximal	39	
Distal	18	
Both	5	
T-stage on MRI		
T3	36	
T4	26	
N-stage on MRI/F18-FDG PET/CT		
N1a/c	25/23	
N1b/c	11/10	
N1c	21	
N1c/2a	5/8	
EMVI grade on MRI		
Grade 2	2	
Grade 3	47	
Grade 4	13	
Number on MRI (patient-based)	TD	LN
1	6	22
2	9	11
3	18	9
4	10	4
5	5	2
>5	14	2
Location on MRI (lesion-based)		
Perirectal	107	76
Superior rectal veins area	106	5
Lateral compartments	0	26

TD: tumor deposit, LN: lymph node (both normal and metastatic).

**Table 2:** Size and distance to nearest high-activity organs for TDs an LNs.

	TD	LN	*p*-Value
Size (mm)	9.0 (3.7)	6.6 (2.4)	<0.001
Distance (mm)	22.5 (16.1)	24.7 (16.8)	0.37

TD: tumor deposit, LN: lymph node (both normal and metastatic).

## Data Availability

Data are contained within the article.
